# HIV prevalence among persons deprived of liberty in Brazil, 2017-2023: a time series analysis

**DOI:** 10.1590/S2237-96222025v34e20240493.en

**Published:** 2025-06-20

**Authors:** Maria Rayssa do Nascimento Nogueira, Hévila Ferreira Gomes Medeiros Braga, Vitória Talya dos Santos Sousa, Nathanael de Souza Maciel, Emanuella Silva Joventino Melo, Patrícia Freire de Vasconcelos, Leilane Barbosa de Sousa

**Affiliations:** 1 Universidade da Integração Internacional da Lusofonia Afro-Brasileira, Programa de Pós-Graduação em Enfermagem, Redenção, CE, Brasil; 2 Universidade Federal do Ceará, Programa de Pós-Graduação em Enfermagem, Fortaleza, CE, Brasil

**Keywords:** HIV, Persons Deprived of Liberty, Health Information Systems, Prevalence, Time Series Analysis, VIH, Personas Privadas de Libertad, Sistemas de Información de Salud, Prevalencia, Estudios de Series de Tiempo

## Abstract

**Objective:**

To analyze the temporal trend of the Human Immunodeficiency Virus (HIV) prevalence in the population deprived of liberty, in Brazil, between the years 2017 and 2023.

**Methods:**

Time series analysis evaluation, from 2017 to 2023. The number of persons deprived of liberty and the number of HIV cases were collected from the. National Penitentiary Department Information System. In addition, sociodemographic variables were collected from each federative unit on the Atlas Brasil website concerning the National Household Sampling Survey (2021). Joinpoint software was used to calculate the annual percentage change (APC) through regressions. The relationship between HIV prevalence and indicators was analyzed in GeoDa 1.22.0.4.

**Results:**

The national prevalence of HIV in persons deprived of liberty was 10.84 cases per 1,000 inmates, with an increasing trend (APC 0.3; 95%CI 0.1;0.6). This rate was higher among women (23.64/1,000 inmates), however, it showed a decreasing trend (APC 0.7; 95%CI -1.2;-0.2). However, although being lower among men (10.15/1,000 inmates), the trend in this group was increasing (APC 0.5; 95%CI 0.2;0.8). As for region, the highest prevalence was in the South (15.12/1,000 inmates). Aging rate (p-value 0.014), per capita household income (p-value 0.021) and municipal human development Index (MHDI) adjusted for income (p-value 0.024) showed positive associations.

**Conclusion:**

Nationally, the prevalence was higher among women. However, while the prevalence among men showed an increasing trend, a reduction was observed among women over the period analyzed.

## Resumen

**Objetivo:**

Analizar la tendencia temporal de la prevalencia del Virus de Inmunodeficiencia Humana (VIH) en la población privada de libertad, en Brasil, entre 2017 y 2023.

**Métodos:**

Estudio de análisis de series de tiempo, de 2017 a 2023. El número de personas privadas de libertad y el número de casos de VIH se recopilaron del Sistema de Información del Departamento Penitenciario Nacional. Además, se recolectaron variables sociodemográficas por unidad federativa en el sitio web Atlas Brasil referente a la Encuesta Nacional por Muestreo de Hogares (2021). Se utilizó el software Joinpoint para calcular el cambio porcentual anual (APV) mediante regresiones. La relación entre la prevalencia del VIH y los indicadores se analizó en GeoDa 1.22.0.4.

**Resultados:**

La prevalencia nacional de VIH en personas privadas de libertad fue de 10,84 casos por cada 1.000 internos, con tendencia creciente (APC 0,3; IC95% 0,1; 0,6). Esta tasa fue mayor entre las mujeres (23,64/1.000 detenidos), sin embargo, mostró una tendencia decreciente (VPA -0,7; IC95% -1,2; -0,2). Sin embargo, aunque menor entre los hombres (10,15/1.000 detenidos), la tendencia en este grupo fue creciente (VPA 0,5; IC95% 0,2; 0,8). En cuanto a la región, la prevalencia más alta se registró en el Sur (15,12/1.000 detenidos). La tasa de envejecimiento (valor-p 0,014), el ingreso familiar per cápita (valor-p 0,021) y el índice de desarrollo humano municipal ajustado por ingreso (valor-p 0,024) mostraron asociaciones positivas.

**Conclusión:**

A nivel nacional la prevalencia fue mayor entre las mujeres. Sin embargo, mientras que la prevalencia entre los hombres mostró una tendencia creciente, se observó una reducción entre las mujeres durante el período analizado.

**Ethical aspects**

This research used public domain and anonymized databases.:

## Introduction

The Human Immunodeficiency Virus (HIV) is a sexually transmitted infection (STI) and the cause of AIDS (acquired immunodeficiency syndrome). Transmission can occur through sexual contact, through blood or vertically (mother to child), and is present in all countries of the world. According to the most recent statistics, 39 million people are living with HIV worldwide (1). In Brazil, it is estimated that one million people are infected with it (2). 

Globally, HIV disproportionately affects certain vulnerable populations, who often face barriers to adequate access to health care and services, including individuals deprived of their liberty. People in prison are 7.2 times more likely to live with the virus than adults in the general population (3). This may be related to several factors, such as overcrowding and inadequate environments, which adds to risky behaviors, such as unprotected sexual intercourse and possible sharing of objects such as syringes and needles, making this population more prone to contracting STI (4). 

The population deprived of liberty in Brazil is defined as “those over the age of 18 (eighteen) and who are in the custody of the State on a provisional basis or sentenced to serve a custodial sentence or security measure” (5). Due to the specific context in which they live, these persons are considered to be highly vulnerable, as there are obstacles to their inclusion in the health care network, in addition to the lack of prioritization of public policies aimed at combating HIV in this population (6).

Globally, it is estimated that 4.2% of persons deprived of liberty are living with HIV, although this prevalence varies considerably between countries (7). In those in development, more worrying scenarios are often reported. In India, for example, 10% of inmates assessed were infected with the virus (8), while in Ethiopia, a survey of almost 10,800 participants found a prevalence of 3.4% (9). In contrast, countries with better sociodemographic conditions have lower prevalence rates: France (2%) and United States (1.1%) (11).

In Brazil, the National Penal Information System revealed that, of the 844,388 people incarcerated, 10,002 were living with HIV in the second half of 2023 (12) However, the analysis by state is still limited and presents significant variations. In Roraima, an investigation identified a prevalence of 4.7% among women deprived of liberty (13), while in Piauí, where both sexes were evaluated, the prevalence was 1% among men and 1.3% among women (6).

From this, it is valid to consider the impact of social determinants of health on the global burden of virus infections. Variables such as male gender, use of illicit drugs, financial hardship and a history of violence have been associated with HIV infection in different studies (14-15). Regarding the population deprived of liberty, sexual practices between same-sex partners - especially between men - stand out, as does the frequent lack of condom use, often due to the lack of access to this method of protection (6).

Therefore, it is essential to reflect on the multiple vulnerability factors related to the prison population, to understand them and offer comprehensive health care. (16) Additionally, it should be noted that no other time series analysis study with the same temporal scope and assessing the entire national territory was identified. Thus, this research is unique, considering the continental dimensions of Brazil and its social, economic and cultural differences, which can also influence how HIV cases are distributed in the population in question, as well as their variations over time. 

Therefore, investigating such contexts becomes relevant to support public and health policies that address the specific needs of each region. Therefore, the aim of this study was to analyze the temporal trend of HIV prevalence in the prison population in Brazil, between 2017 and 2023.

## Methods

### Study design

Time series analysis study, prepared in accordance with the recommendations of *The Reporting of Studies Conducted Using Observational Routinely Collected Health Data* (17).

### Context 

The prevalence of HIV in the prison population in the 27 Federative Units of Brazil was analyzed. Data collection took place between June and July of 2024. 

### Variables 

Data relating to cycles 2 (2017.1) to 15 (2023.2) were used, from which the following variables were collected: number of persons deprived of liberty and number of HIV cases in persons deprived of liberty. These variables were grouped by sex (male and female), federation unit and region, as well as the total values. The time frame was adopted because these were the years for which the data for the variables mentioned were complete, with data from the first and second semesters. Thus, to calculate prevalence, the number of HIV cases per location in the period was considered, divided by the total number of persons deprived of liberty in the same location and period, multiplied by a thousand.

In addition, sociodemographic variables were collected by federative unit on the Atlas Brazil referring to the National Household Sampling Survey - 2021 website. Socioeconomic indicators included Municipal human development index (MHDI), Aging rate, Percentage of people vulnerable to poverty, Per capita income, Illiteracy rate for people aged 25 or older and Inequality-adjusted income.

### Data sources

The information was obtained from the National Penitentiary Department Information System Database, a web system developed to collect and store penitentiary data nationwide. (18) This data source was used because the data from the Notifiable Diseases Information System, made available by TabNet, records general data on HIV/AIDS notifications throughout the country, but does not allow for the direct segmentation of detailed information about the population deprived of liberty. From Microsoft Excel spreadsheets made available in open access (https://www.gov.br/senappen/pt-br/servicos/sisdepen/bases-de-dados), the variables were filtered by year of interest using the software’s native tool. From this, the values per prison unit were added together, thus forming the values per state, region and total for the country.

### Statistical methods

The data available online were downloaded in CVS (Comma Separated Values) format and organized into a single Google Sheets spreadsheet. To analyze the temporal trend of prevalence, the Joinpoint Regression Program software version 5.1.0 was used to conduct a segmented regression analysis (inflection points) (19. This method allows us to examine whether there are variations in the prevalence trend over time, providing an estimate of the average annual percentage change (APC), expressing the change in percentage terms from one year to the next, together with its 95% Confidence Interval (95%CI), based on a statistical significance criterion established at a p-value<0.05. When APC is negative, it indicates a decreasing trend over time; on the other hand, positive values suggest an increasing trend. Finally, if the p-value<0.05, the APC is considered not statistically significant, indicating an absence of variation in the trend, i.e., a stationary trend (20).

The relationship between HIV prevalence and indicators was analyzed using the GeoDa 1.22.0.4 software, with the non-spatial regression model called Ordinary Least Squares. β coefficients and p-values were calculated for each variable, with those with a p-value<0.05 indicating statistical significance.

## Results

**Figure 1:**
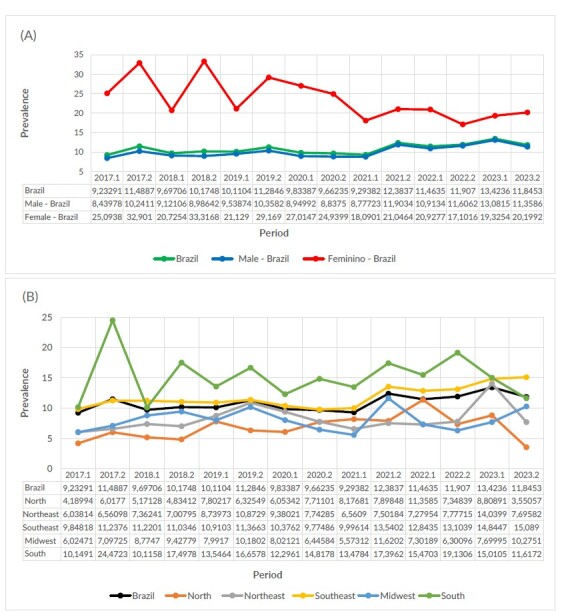
Temporal trend in the prevalence of the Human Immunodeficiency Virus (HIV) in persons deprived of liberty in Brazil between 2017 and 2023, at the national level, according to gender (A) and region (B). Brazil, 2017-2023

Between 2017 and 2023, the prevalence of HIV among persons deprived of liberty in Brazil was 10.84 cases per 1,000 inmates. This rate was higher among women (23.64/1,000 inmates) than among men (10.15/1,000 inmates). In relation to the region of the country, the highest prevalence was identified in the South (15.12/1,000 inmates), followed by Southeast (11.80/1,000), Northeast (8.18/1,000), Midwest (8.05/1,000) and North (6.80/1,000) (Figure 1).

Regarding the states, the highest prevalence was identified in Rio Grande do Sul (27.37/1,000), Santa Catarina (20.4827.37/1,000), São Paulo (13.6727.37/1,000) Rio de Janeiro (13.3327.37/1,000) Pernambuco (12.5727.37/1,000) and Bahia (11.8327.37/1,000), both above the national prevalence. The states with the lowest prevalence were: Acre (1.30/1,000), Amapá (2.40/1,000), Alagoas (3.65/1,000), Maranhão (3.78/1,000), Minas Gerais (5.09/1,000) and Tocantins (5.35/1,000).

During this period, the trend in HIV prevalence among persons deprived of liberty in Brazil varied by sex and region. At the national level, HIV prevalence showed an increasing trend (APC 0.3; 95%CI 0.1; 0.6). Among men, the trend was also increasing (APC 0.5; 95%CI 0.2; 0.8). In contrast, among women, HIV prevalence showed a decreasing trend (APC -0.7; 95%CI -1.2; -0.2).

In terms of regions, the analysis shows that in the Southeast, the trend in HIV prevalence was increasing both in the total population (APC 0.5; 95%CI 0.2; 0.8) and among men (VPA 0.7; 95%CI 0.3; 1.0). In the Northeast, a decreasing trend was observed among women (APC -0.8; 95%CI -1.3; -0.3) (Table 1).

**Table 1:** Annual percentage change (APC) and 95% confidence intervals (95%CI) of the prevalence of the Human Immunodeficiency Virus (HIV) in persons deprived of liberty, by sex and region. Brazil, 2017-2023

Location	APC (95%CI)	p-value	Trend
Brazil	0.3 (0.1;0.6)	0.024	Growing
Male	0.5 (0.2;0.8)	0.006	Growing
Female	-0.7 (-1.2;-0.2)	0.006	Decreasing
North	0.6 (-0.3;1.5)	0.173	Stationary
Male^a^	0.6 (-0.3;1.5)	0.168	Stationary
Female	-0.4 (-2.7;2.0)	0.752	Stationary
Northeast	0.5 (-0.2;1.1)	0.135	Stationary
Male	0.5 (-0.2;1.1)	0.174	Stationary
Female	-0.8 (-1.3;-0.3)	0.003	Decreasing
Southeast	0.5 (0.2;0.8)	0.004	Growing
Male	0.7 (0.3;1.0)	0.001	Growing
Female	-0.5 (-1.3;0.2)	0.150	Stationary
Midwest	0.1 (-0.6;0.8)	0.774	Stationary
Male	0.0 (-0.6;0.7)	0.944	Stationary
Female	0.2 (-1.0;1.5)	0.691	Stationary
South	0.0 (-0.7;0.8)	0.956	Stationary
Male	0,3 (-0.4;1.1)	0.381	Stationary
Female^b^	-1.4 (-3.0;0.2)	0.073	Stationary

^a^Data on HIV cases in men in the North in 2021.1 were excluded, as a discrepant value (outlier) was found; ^b^Data on HIV cases in women in the South in 2018.2 were excluded, as a discrepant value (outlier) was found.

Between 2017 and 2023, the trend in HIV prevalence among persons deprived of liberty varied significantly by federative unit in Brazil. In the North, the federative units with increasing trends were Pará (APC 1.5; 95%CI 0.7; 2.2), Rondônia (APC 1.3; 95%CI, 0.4; 2.3), Roraima (APC 1.1; 95%CI 0.4; 1.8) and Tocantins (APC 0.7; 95%CI 0.2;1.3).

In the Northeast, Maranhão (APC 1.9; 95%CI 1.0; 2.8) and Rio Grande do Norte (APC 1.7; 95%CI 1.1; 2.3) showed increasing trends in HIV prevalence, while Pernambuco showed a decreasing trend (APC -1.1; 95%CI -1.8; -0.3). In the Southeast, Rio de Janeiro stood out with a growing trend (APC 3.2; 95%CI 2.2; 4.2). In the South, Paraná showed a decreasing trend in HIV prevalence (APC -1.4; 95%CI -2.2; -0.5) (Table 2).

**Table 2:** Annual percentage change (APC) and 95% confidence intervals (95%CI) of the prevalence of the Human Immunodeficiency Virus (HIV) in persons deprived of liberty, by Federative Unit. Brazil, 2017-2023

Location	APC (95%CI)	p-value	Trend
North			
Acre	-0.5 (-2.4;1.5)	0.605	Stationary
Amapá	2.0 (-1.3;5.4)	0.224	Stationary
Amazonas	1.1 (-0.3;2.6)	0.102	Stationary
Pará	1.5 (0.7;2.2)	0.001	Growing
Rondônia	1.3 (0.4;2.3)	0.008	Growing
Roraima	1.1 (0.4;1.8)	0.005	Growing
Tocantins	0.7 (0.2;1.3)	0.009	Growing
Northeast			
Alagoas	0.4 (-0.6;1.4)	0.383	Stationary
Bahia	0.9 (-0.1;1.9)	0.081	Stationary
Ceara^a^	0.8 (-1.1;2.7)	0.380	Stationary
Maranhão^b^	1.9 (1.0;2.8)	0.002	Growing
Paraíba	-0.9 (-2.1;0.3)	0.126	Stationary
Pernambuco	-1.1 (-1.8;-0.3)	0.007	Decreasing
Piauí	-0.2 (-1.2;0.7)	0.631	Stationary
Rio Grande do Norte	1.7 (1.1;2.3)	0.001	Growing
Sergipe	0.7 (-0.2;1.6)	0.116	Stationary
Southeast			
Espírito Santo	0.1 (-1.0;1.30)	0.830	Stationary
Minas Gerais^c^	-0.8 (-4.0;2.5)	0.600	Stationary
Rio de Janeiro	3.2 (2.2;4.2)	0.001	Growing
São Paulo	-0.0 (-0.2;0.2)	0.970	Stationary
South			
Paraná	-1.4 (-2.2;-0.5)	0.005	Decreasing
Rio Grande do Sul	1.2 (-0.5;2.9)	0.141	Stationary
Santa Catarina	0.2 (-0.3;0.7)	0.419	Stationary
Midwest			
Distrito Federal	-0.2 (-1.4;1.1)	0.761	Stationary
Goiás	0.1 (-1.4;1.6)	0.864	Stationary
Mato Grosso	-0.5 (-0.9;0.0)	0.054	Stationary
Mato Grosso do Sul	0.5 (-0.4;1.5)	0.258	Stationary

^a^The analysis of data from Ceará begins in 2019, due to unavailability of data and discrepant values (outliers); ^b^Data from Maranhão available from 2019.1; ^c^Minas Gerais did not provide data for the period 2020.2.

Several socioeconomic indicators showed significant association with the prevalence of HIV in persons deprived of liberty in Brazil. The MHDI showed a significant negative association (p-value 0.027), indicating that an increase in the MHDI is associated with a reduction in HIV prevalence. 

The aging rate showed a significant positive association with HIV prevalence (p-value 0.014), indicating that an increase in this rate is associated with an increase in HIV prevalence. 

The percentage of people vulnerable to poverty showed a positive association, but with no statistical significance (p-value 0.056), suggesting that an increase in the proportion of poor people may be associated with an increase in HIV prevalence. 

Per capita household income also showed a significant positive association with HIV prevalence (p-value 0.021), indicating that an increase in per capita household income is associated with an increase in HIV prevalence. In contrast, the illiteracy rate for people aged 25 and over showed a significant negative association (p-value 0.035), suggesting that an increase in the illiteracy rate is associated with a reduction in HIV prevalence.

Finally, income-adjusted MHDI showed a significant positive association with HIV prevalence (p-value 0.024), indicating that an increase in income-adjusted MHDI is associated with an increase in HIV prevalence (Table 3).

**Table 3:** Association between the prevalence of the Human Immunodeficiency Virus (HIV) in persons deprived of liberty and socioeconomic indicators. Brazil, 2017-2023

Socioeconomic indicators	Coefficient	Error	p-value
Municipal human development index	-186.67	77.76	0.027
Aging rate	1.28	0.47	0.014
Percentage of people vulnerable to poverty	0.74	0.36	0.056
Per capita income	0.02	0.01	0.021
Illiteracy rate^a^	-0.60	0.26	0.035
Inequality-adjusted income	143.92	58.45	0.024

^a^Aged 25 years or older.

## Discussion

Between 2017 and 2023, the prevalence of HIV among persons deprived of liberty in Brazil revealed striking patterns of inequality by sex, region and federative unit, in addition to association with socioeconomic indicators. The prevalence rate was higher among women, although the trend over the period was decreasing, in contrast to the increasing trend observed among men.

The South and Southeast regions recorded the highest prevalence, with emphasis on Rio Grande do Sul State. However, the analysis of trends pointed to significant variations between regions and states: Pará and Rio de Janeiro showed an increase, while Paraná and Pernambuco saw a reduction.

It is worth noting that the prevalence of HIV in the prison environment is higher when compared to the general population (21). In this context, in addition to the unhealthy and overcrowded conditions of these environments, the sociodemographic profile of persons deprived of liberty favors an increased vulnerability to HIV and other infectious diseases (6).

The prevalence of HIV infection in the prison environment reflects several structural and social factors, such as the lack of adequate access to health care and the precarious living conditions within prisons, which facilitate the spread of the virus (22). Furthermore, unsafe sexual practices, mainly due to violence, drug use and sharing of sharp objects, are among the main risks that determine the health needs of this population (6).

The stigmatization and marginalization of this population can also hinder access to prevention and treatment programs, increasing their vulnerability to HIV (23). The spread of HIV in prisons is also closely dependent of effective health policies and specific interventions that address the particularities of this context in different regions (24).

A higher HIV prevalence rate was found in Brazil among women deprived of liberty, compared to men, which suggests that women face specific and more complex challenges in the prison environment. Therefore, a combination of vulnerability factors may be related not only to the lack of access to information on prevention and transmission methods, but also to exposure to sexual violence, distorted perceptions of risk or difficulty in obtaining condoms and assistance from health professionals (13,25). The high prevalence may also reflect under diagnosis in previous periods, greater recent screening and the impact of the prison unit as a location of high transmission.

However, even with the high HIV prevalence rate in Brazil among women in the prison system, the trend in this prevalence was decreasing over the period of time analyzed in this study. In view of this, the Brazilian National Policy of Attention to Women Deprived of Liberty and Released from the Prison System, enacted in 2014, stands out, aimed at meeting the specific needs of women and ensuring humanization of the conditions for serving sentences, although studies show a lack of effectiveness in complying with this policy, considering that institutional barriers, overcrowding and discrimination affect the right of these women to health care (26-27).

This study revealed a higher prevalence of HIV in the South and Southeast regions of Brazil. Despite the economic development of these regions, vulnerable populations may face barriers in accessing adequate health care. High population density in urban environments, especially in low-income areas, can lead to risky practices, such as the use of injecting drugs and unprotected sexual intercourse, which are risk factors for HIV transmission (28). 

During the period evaluated by this study, trends in HIV prevalence in the prison population varied between regions and states. In the Southeast, an increase in the trend was observed, mainly among men. These results are consistent with data from a study on the prevalence of AIDS in the Southeast region, which identified a predominance of infection in the male population, with emphasis on the states of Rio de Janeiro and São Paulo (29).

On the other hand, states such as Pernambuco and Paraná showed a downward trend in HIV prevalence, which may indicate progress in combating the disease. Thus, the identification of HIV-positive inmates makes possible to formulate prevention strategies, such as expanding rapid testing and health education, in addition to adequate access to health services for treatment and ongoing monitoring, which may have contributed to reducing transmission of the virus among the prison population in these states (30). 

Expanding access to HIV diagnosis through rapid testing favors early detection and timely treatment, in addition to the fact that coordination with the health care network is essential to offer comprehensive care and contribute to suppressing the viral load of people living with HIV in the prison system (23). Such measures, when implemented, can explain the stationary pattern of HIV prevalence trends in most Brazilian states and regions. 

An association was also observed between the increase in the MHDI and the reduction in the HIV infection rate among persons deprived of liberty. The MHDI is a measure that analyzes the human development of a municipality in terms of income, education and health. In fact, analyses of global secondary data, provided by the Global Burden of Disease Study, indicate that between 1990 and 2019, regions with greater socioeconomic development showed a decrease in these rates (31). In line with this, among persons deprived of liberty, it is observed that in cities in South Africa, a country considered the epicenter (32) of the HIV/AIDS epidemic, the incidence of HIV seropositivity cases has increased and shows a prevalence of 17.7% (33), while in Europe, in cities of countries such as France and Italy, the prevalence varies from ≥2% to <5%, respectively (34).

However, the relationship between MHDI, income and HIV prevalence may vary spatially and temporally within a country due to regional differences, which may have a positive or negative association, regardless of whether the context is favorable or not (35). When it comes to the general population, it was observed that this scenario also occurs. In this study, for example, increases in national income, increases in income-adjusted MHDI, and in the proportion of poor individuals are associated with increases in HIV prevalence among persons deprived of liberty. 

Specifically regarding income, it is understood that it is not feasible to establish a comparison between the economic situation of the population deprived of liberty and HIV seropositivity, since, when participating in research, many choose not to report their income, fearing that this information could be linked to the crime or offense committed, in addition to which, the National Penitentiary Information Survey system does not provide data on the income of the prison population in Brazil (28).

The relationship between the increase in individuals living in poverty and the incidence of HIV may be related to risky sexual behaviors. A study carried out in Malawi, an African country with one of the lowest Human Development Indexes (HDI) in the world (0.0512) (36) and an HIV prevalence of over 23% among the prison population (37), revealed that poverty led some inmates to engage in sexual activities in exchange for benefits, such as sleeping space and better food, provided by prisoners with longer sentences (38).

The illiteracy rate among people aged 25 and over showed a negative relationship with the prevalence of HIV, suggesting that states with more people who cannot read or write register fewer cases of HIV. However, it is believed that this association may reflect underreporting of data, due to low adherence to diagnostic strategies among this population, since, as they have low health literacy, they may be unaware of HIV diagnostic measures, which may influence whether they take the test or not (39).

Furthermore, the increasing aging rate of HIV prevalence among the prison population may be linked to higher use of HIV screening tests, reflecting greater awareness of HIV risks among older people compared to young people in prison (39–40).

As a limitation of the study, the source of the data analyzed stands out. They were obtained in a system that may be subject to underreporting and inadequacy of records, which may compromise the quality of the information collected. Underreporting may occur due to the lack of systematic testing and difficulties in adequately recording cases, impacting the real dimension of HIV prevalence in this population. 

Furthermore, the use of data from the penitentiary system, to the detriment of other databases, such as SINAN (The Notifiable Diseases Information System), may not cover all prison units in the country uniformly, especially in states or regions with less penitentiary management capacity. This system may not integrate information prior to or after the period of the deprivation of liberty, such as diagnoses carried out in external health units, limiting the analysis of the clinical trajectory of persons deprived of liberty. 

Another important limitation arises from the time series design, which allows the identification of patterns and trends over time, but does not allow the establishment of causal relationships or individual extrapolations. The interpretation of the results must consider these aspects, and future research can deepen these analyses with complementary methodological approaches.

This study highlighted significant variations in the temporal trend of HIV prevalence by sex, region and state among the Brazilian population deprived of liberty. Nationally, the prevalence was higher among women, but showed a downward trend over the period analyzed, while there was an increase among men. In the Southeast, HIV prevalence increased among men, while the Northeast recorded a reduction among women. In states such as Rio de Janeiro and Pará, the prevalence increased among men, while in Paraná and Pernambuco, the downward prevalence trend was greater among women. The drop in the number of cases among women and the increase among men indicates the need for effective and targeted actions through the implementation of preventive and treatment strategies among the male population, which must be organized strategically, considering the size of this population.

Findings on socioeconomic determinants, such as the income-adjusted municipal human development index, per capita household income and illiteracy rates, were shown to be associated with HIV prevalence, highlighting the influence of structural inequalities. These results point to the complexity of the scenario and the need for integrated approaches that combine public health actions with initiatives to reduce social inequalities.

The presentation of this panorama can, therefore, contribute to the strategic modulation of health actions, indicating that their planning should be based on regional and gender disparities, and that they should consider socioeconomic determinants when formulating their interventions to reduce the prevalence of HIV in persons deprived of liberty in Brazil.

## Data Availability

The database used in the research is available at https://osf.io/shn7k/.
