# Supplementary material for: HIV prevalence among persons deprived of liberty in Brazil, 2017-2023: a time series analysis
Source: Epidemiol Serv Saude. 2025 Jun 20;34:e20240493. doi: 10.1590/S2237-96222025v34e20240493.en (PMC12204062; doi:10.1590/S2237-96222025v34e20240493.en)
Supplement: Translation

# Translation: Prevalência do HIV em pessoas privadas de liberdade no Brasil, 2017-2023: análise de série temporal

**DOI:** 10.1590/S2237-96222025v34e20240493.pt

**Authors:** Maria Rayssa do Nascimento Nogueira, Hévila Ferreira Gomes Medeiros Braga, Vitória Talya dos Santos Sousa, Nathanael de Souza Maciel, Emanuella Silva Joventino Melo, Patrícia Freire de Vasconcelos, Leilane Barbosa de Sousa

**Affiliations:** 1 Universidade da Integração Internacional da Lusofonia Afro-Brasileira, Programa de Pós-Graduação em Enfermagem, Redenção, CE, Brasil; 2 Universidade Federal do Ceará, Programa de Pós-Graduação em Enfermagem, Fortaleza, CE, Brasil

**Keywords:** HIV, Pessoas Privadas de Liberdade, Sistemas de Informação em Saúde, Prevalência, Estudos de Séries Temporais

## Abstract

**Objetivo:**

analisar a tendência temporal da prevalência do Vírus da Imunodeficiência Humana (HIV) na população privada de liberdade, no Brasil, entre os anos de 2017 e 2023.

**Métodos:**

estudo de análise de série temporal, do período de 2017 a 2023. Do Sistema de Informações do Departamento Penitenciário Nacional foi coletado o número de pessoas privadas de liberdade e número de casos de HIV. Além disso, foram coletadas variáveis sociodemográficas por unidade federativa no site Atlas Brasil referente à Pesquisa Nacional de amostragem por domicílio (2021). Utilizou-se o software Joinpoint para calcular a variação percentual anual (VPA) por meio de regressões. A relação entre prevalência de HIV e os indicadores foi analisada no GeoDa 1.22.0.4.

**Resultados:**

a prevalência nacional do HIV em pessoas privadas de liberdade foi de 10,84 casos por 1.000 detentos, com uma tendência crescente (VPA 0,3; IC95% 0,1; 0,6). Essa taxa foi maior entre as mulheres (23,64/1.000 detentos), porém, mostrou uma tendência decrescente (VPA -0,7; IC95% -1,2; -0,2). Porém, ainda que menor entre os homens (10,15/1.000 detentos), a tendência nesse grupo foi crescente (VPA 0,5; IC95% 0,2; 0,8). Quanto à região, a maior prevalência foi no Sul (15,12/1.000 detentos). Taxa de envelhecimento (p-valor 0,014), renda domiciliar per capita (p-valor 0,021) e índice de desenvolvimento humano municipal ajustado para renda (p-valor 0,024) mostraram associações positivas.

**Conclusão:**

nacionalmente, a prevalência foi maior entre as mulheres. Contudo, enquanto a prevalência entre os homens apresentou tendência crescente, entre as mulheres foi observada redução ao longo do período analisado.

**Aspectos éticos**

Esta pesquisa utilizou bancos de dados de domínio público e anonimizados.:

## Introdução

O Vírus da Imunodeficiência Humana (HIV) é uma infecção sexualmente transmissível (IST) e o causador da aids. A transmissão pode acontecer por via sexual, sanguínea ou vertical, e está presente em todos os países do mundo. Segundo as estatísticas mais recentes, 39 milhões de pessoas vivem com HIV no mundo (1). No Brasil, estima-se que o número chegue a um milhão de infectados (2). 

O HIV afeta desproporcionalmente certas populações vulneráveis em nível global, que enfrentam, muitas vezes, barreiras para o acesso adequado às ações e serviços de saúde, o que inclui indivíduos privados de liberdade. Pessoas na prisão têm 7,2 vezes mais chances de viver com o vírus que adultos da população geral (3). Isso pode estar relacionado a diversos fatores, como a superlotação e ambientes inadequados, o que se soma a comportamentos de risco, como relações sexuais desprotegidas e possível compartilhamento de objetos como seringas e agulhas, tornando esse público mais propenso a contrair IST (4). 

Define-se como população privada de liberdade, no Brasil, “aquelas com idade superior a 18 (dezoito) anos e que estejam sob a custódia do Estado em caráter provisório ou sentenciados para cumprimento de pena privativa de liberdade, ou medida de segurança” (5). Devido ao contexto específico na qual estão inseridas, considera-se que essas pessoas se encontram em alta vulnerabilidade, pois existem obstáculos na sua inclusão na rede de atenção à saúde, além da não priorização de políticas públicas voltadas para o enfrentamento do HIV nesse público (6).

Globalmente, estima-se que 4,2% das pessoas privadas de liberdade vivem com HIV, embora essa prevalência varie consideravelmente entre os países (7). Naqueles em desenvolvimento, cenários mais preocupantes são frequentemente relatados. Na Índia, por exemplo, 10% dos detentos avaliados estavam infectados pelo vírus (8), enquanto na Etiópia, uma pesquisa com quase 10.800 participantes apontou uma prevalência de 3,4% (9). Em contrapartida, países com melhores condições sociodemográficas apresentam prevalências menores: França (2%) e Estados Unidos (1,1%) (11).

No Brasil, o Sistema Nacional de Informações Penais revelou que, no segundo semestre de 2023, das 844.388 pessoas encarceradas, 10.002 viviam com HIV (12). Entretanto, a análise por estado ainda é limitada e apresenta variações significativas. Em Roraima, uma investigação identificou uma prevalência de 4,7% entre mulheres privadas de liberdade (13), enquanto no Piauí, onde ambos os sexos foram avaliados, a prevalência foi de 1% entre homens e 1,3% entre mulheres (6).

A partir disso, é válido considerar o impacto dos determinantes sociais em saúde na carga global de infecções pelo vírus. Variáveis como sexo masculino, uso de drogas ilícitas, dificuldades financeiras e histórico de violência foram associados à infecção do HIV em diferentes pesquisas (14-15). No que concerne à população privada de liberdade, destaca-se a prática sexual entre parceiros do mesmo sexo - especialmente entre homens - e, a frequente ausência do uso de preservativos, muitas vezes devido à falta de acesso a esse método de proteção (6).

Assim, refletir sobre os múltiplos fatores de vulnerabilidade relacionados à população prisional torna-se essencial para compreendê-los e oferecer um cuidado integral em saúde (16). Em complemento, salienta-se que não foram identificados outros estudos de análise de série temporal com a mesma abrangência temporal e que considerassem todo o território nacional. Desta forma, esta pesquisa diferencia-se ao considerar as dimensões continentais do Brasil e suas diferenças sociais, econômicas e culturais, o que também pode influenciar em como estão dispostos os casos de HIV na população em questão, bem como suas variações ao longo do tempo. 

Assim, investigar tais contextos torna-se relevante para apoiar políticas públicas e de saúde que contemplem as necessidades específicas de cada região. Portanto, objetivou-se analisar a tendência temporal da prevalência do HIV na população privada de liberdade, no Brasil, entre os anos de 2017 e 2023.

## Métodos

### Delineamento do estudo

Estudo de análise de série temporal, redigido de acordo com as recomendações do *The Reporting of Studies Conducted Using Observational Routinely Collected Health Data* (17).

### Contexto 

Analisou-se a prevalência do HIV na população privada de liberdade nas 27 Unidades Federativas. A coleta de dados ocorreu entre junho e julho de 2024. 

### Variáveis 

Foram utilizados dados referentes aos ciclos 2 (2017.1) a 15 (2023.2), dos quais foram coletadas as variáveis: número de pessoas privadas de liberdade e número de casos de HIV em pessoas privadas de liberdade. Essas variáveis foram agrupadas por sexo (masculino e feminino), Unidade da Federação e região, bem como os valores totais. Adotou-se o recorte temporal, pois eram os anos em que os dados das variáveis citadas estavam completos, com dados do primeiro e segundo semestre. Assim, para o cálculo da prevalência foi considerado o número de casos de HIV por local no período, dividido pelo total de pessoas privadas de liberdade do mesmo local e período, multiplicado por mil.

Além disso, foram coletadas variáveis sociodemográficas por unidade federativa no site do Atlas Brasil referente à Pesquisa Nacional de Amostragem por Domicílio, 2021. Os indicadores socioeconômicos incluíram índice de desenvolvimento humano municipal (IDHM), Taxa de envelhecimento, Percentual de vulneráveis à pobreza, Renda per capita, Taxa de analfabetismo - 25 anos ou mais de idade e Renda ajustado à desigualdade.

### Fontes de dados

As informações foram obtidas no banco de dados do Sistema de Informações do Departamento Penitenciário Nacional, um sistema web desenvolvido para coletar e armazenar dados penitenciários em âmbito nacional (18). Essa fonte de dados foi utilizada, pois os dados do Sistema de Informação de Agravos de Notificação, disponibilizados pelo TabNet registra dados gerais de notificações de HIV/aids em todo o país, mas não permite segmentar diretamente informações detalhadas sobre a população privada de liberdade. A partir de planilhas do Microsoft Excel disponibilizadas em acesso aberto (https://www.gov.br/senappen/pt-br/servicos/sisdepen/bases-de-dados), filtrou-se, por meio de ferramenta nativa do software, as variáveis por ano de interesse. A partir disso, foram somados os valores por unidade prisional, formando-se assim os valores por estado, região e total do país.

### Métodos estatísticos

Os dados disponíveis online foram baixados no formato valores separados por vírgulas e organizados em uma única planilha do Google Sheets. Para a análise da tendência temporal da prevalência, utilizou-se o software Joinpoint Regression Program versão 5.1.0 para conduzir uma análise de regressão segmentada (pontos de inflexão) (19). Esse método permite examinar se há variações na tendência da prevalência ao longo do tempo, fornecendo uma estimativa da variação percentual anual média (VPA), expressando a mudança em termos percentuais de um ano para o seguinte, juntamente com intervalo de confiança de 95% (IC95%), com base em um critério de significância estatística estabelecido em p-valor<0,05. Quando a VPA é negativa, isso indica uma tendência decrescente ao longo do tempo; por outro lado, valores positivos sugerem uma tendência crescente. Por fim, se o p-valor<0,05, a VPA não é considerada estatisticamente significativa, indicando uma ausência de variação na tendência, ou seja, uma tendência estacionária (20).

A relação entre prevalência de HIV e indicadores foi analisada no software GeoDa 1.22.0.4, por meio do modelo de regressão não espacial chamado Ordinary Least Squares. Foram calculados coeficientes β e valores de p-valor para cada variável, indicando significância estatística aqueles com p-valor<0,05.

## Resultados

Entre 2017 e 2023, a prevalência do HIV em pessoas privadas de liberdade no Brasil foi de 10,84 casos por 1.000 detentos. Essa taxa foi maior entre as mulheres (23,64/1.000 detentos) do que em homens (10,15/1.000 detentos). Em relação à região do país, a maior prevalência foi identificada no Sul (15,12/1.000 detentos), seguido do Sudeste (11,80/1.000), Nordeste (8,18/1.000), Centro-Oeste (8,05/1.000) e Norte (6,80/1.000) (Figura 1).

**Figura 1:** Tendência temporal da prevalência de HIV em pessoas privadas de liberdade, de acordo com o gênero (A) e região (B). Brasil, 2017-2023

Quanto aos estados, maior prevalência foi identificada no Rio Grande do Sul (27,37/1.000), Santa Catarina (20,4827,37/1.000), São Paulo (13,6727,37/1.000) Rio de Janeiro (13,3327,37/1.000) Pernambuco (12,5727,37/1.000) e Bahia (11,8327,37/1.000), ambos acima da prevalência nacional. Os estados de menor prevalência foram: Acre (1,30/1.000), Amapá (2,40/1.000), Alagoas (3,65/1.000), Maranhão (3,78/1.000), Minas Gerais (5,09/1.000) e Tocantins (5,35/1.000).

No período, a tendência da prevalência de HIV em pessoas privadas de liberdade no Brasil variou por sexo e região. No nível nacional, a prevalência de HIV mostrou uma tendência crescente (VPA 0,3; IC95% 0,1; 0,6). Entre os homens, a tendência também foi crescente (VPA 0,5; IC95% 0,2; 0,8). Em contraste, entre as mulheres, a prevalência de HIV mostrou uma tendência decrescente (VPA -0,7; IC95% -1,2; -0,2).

Analisando por regiões, no Sudeste, a tendência da prevalência de HIV foi crescente tanto na população total (VPA 0,5; IC95% 0,2; 0,8), quanto entre os homens (VPA 0,7; IC95% 0,3; 1,0). No Nordeste, observou-se uma tendência decrescente entre as mulheres (VPA -0,8; IC95% -1,3; -0,3) (Tabela 1).

**Tabela 1:** Variação percentual anual (VPA) e intervalos de confiança de 95% (IC95%) da prevalência do HIV em pessoas privadas de liberdade, por sexo e região. Brasil, 2017-2023

Local	VPA (IC95%)	p-valor	Tendência
Brasil	0,3 (0,1; 0,6)	0,024	Crescente
Masculino	0,5 (0,2; 0,8)	0,006	Crescente
Feminino	-0,7 (-1,2; -0,2)	0,006	Decrescente
Norte	0,6 (-0,3; 1,5)	0,173	Estacionária
Masculino^a^	0,6 (-0,3; 1,5)	0,168	Estacionária
Feminino	-0,4 (-2,7; 2,0)	0,752	Estacionária
Nordeste	0,5 (-0,2; 1,1)	0,135	Estacionária
Masculino	0,5 (-0,2; 1,1)	0,174	Estacionária
Feminino	-0,8 (-1,3; -0,3)	0,003	Decrescente
Sudeste	0,5 (0,2; 0,8)	0,004	Crescente
Masculino	0,7 (0,3; 1,0)	0,001	Crescente
Feminino	-0,5 (-1,3; 0,2)	0,150	Estacionária
Centro-Oeste	0,1 (-0,6; 0,8)	0,774	Estacionária
Masculino	0,0 (-0,6; 0,7)	0,944	Estacionária
Feminino	0,2 (-1,0; 1,5)	0,691	Estacionária
Sul	0,0 (-0,7; 0,8)	0,956	Estacionária
Masculino	0,3 (-0,4; 1,1)	0,381	Estacionária
Feminino^b^	-1,4 (-3,0; 0,2)	0,073	Estacionária

^a^Excluído dado de casos de HIV em homens no Norte em 2021.1, por apresentar valor discrepante; ^b^Excluído dado de casos de HIV em mulheres no Sul em 2018.2, por apresentar valor discrepante.

Entre 2017 e 2023, a tendência da prevalência de HIV em pessoas privadas de liberdade variou significativamente por unidade federativa no Brasil. No Norte, as unidades federativas com tendências crescentes foram Pará (VPA 1,5; IC95% 0,7; 2,2), Rondônia (VPA: 1,3; IC95% 0,4; 2,3), Roraima (VPA 1,1; IC95% 0,4;1,8) e Tocantins (VPA 0,7; IC95% 0,2; 1,3).

No Nordeste, Maranhão (VPA 1,9; IC95% 1,0; 2,8) e Rio Grande do Norte (VPA 1,7; IC95% 1,1; 2,3) mostraram tendências crescentes na prevalência de HIV, enquanto Pernambuco apresentou uma tendência decrescente (VPA -1,1; IC95% -1,8; -0,3). No Sudeste, o Rio de Janeiro destacou-se com uma tendência crescente (VPA 3,2; IC95% 2,2; 4,2). No Sul, Paraná apresentou uma tendência decrescente na prevalência de HIV (VPA -1,4; IC95% -2,2; -0,5) (Tabela 2).

**Tabela 2:** Variação percentual anual (VPA) e intervalos de confiança de 95% (IC95%) da prevalência do HIV em pessoas privadas de liberdade, por Unidade Federativa. Brasil, 2017-2023

Local	VPA (IC95%)	p-valor	Tendência
Norte			
Acre	-0,5 (-2,4; 1,5)	0,605	Estacionária
Amapá	2,0 (-1,3; 5,4)	0,224	Estacionária
Amazonas	1,1 (-0,3; 2,6)	0,102	Estacionária
Pará	1,5 (0,7; 2,2)	0,001	Crescente
Rondônia	1,3 (0,4; 2,3)	0,008	Crescente
Roraima	1,1 (0,4; 1,8)	0,005	Crescente
Tocantins	0,7 (0,2; 1,3)	0,009	Crescente
Nordeste			
Alagoas	0,4 (-0,6; 1,4)	0,383	Estacionária
Bahia	0,9 (-0,1; 1,9)	0,081	Estacionária
Ceará^a^	0,8 (-1,1; 2,7)	0,380	Estacionária
Maranhão^b^	1,9 (1,0;2,8)	0,002	Crescente
Paraíba	-0,9 (-2,1;0,3)	0,126	Estacionária
Pernambuco	-1,1 (-1,8; -0,3)	0,007	Decrescente
Piauí	-0,2 (-1,2; 0,7)	0,631	Estacionária
Rio Grande do Norte	1,7 (1,1; 2,3)	0,001	Crescente
Sergipe	0,7 (-0,2; 1,6)	0,116	Estacionária
Sudeste			
Espírito Santo	0,1 (-1,0; 1,30)	0,830	Estacionária
Minas Gerais^c^	-0,8 (-4,0; 2,5)	0,600	Estacionária
Rio de Janeiro	3,2 (2,2; 4,2)	0,001	Crescente
São Paulo	-0,0 (-0,2; 0,2)	0,970	Estacionária
Sul			
Paraná	-1,4 (-2,2; -0,5)	0,005	Decrescente
Rio Grande do Sul	1,2 (-0,5; 2,9)	0,141	Estacionária
Santa Catarina	0,2 (-0,3; 0,7)	0,419	Estacionária
Centro-Oeste			
Distrito Federal	-0,2 (-1,4; 1,1)	0,761	Estacionária
Goiás	0,1 (-1,4;1,6)	0,864	Estacionária
Mato Grosso	-0,5 (-0,9;0,0)	0,054	Estacionária
Mato Grosso do Sul	0,5 (-0,4;1,5)	0,258	Estacionária

^a^A análise dos dados do Ceará inicia-se em 2019, por indisponibilidade de dados e valores discrepantes; ^b^Dados do Maranhão disponíveis a partir de 2019.1; ^c^Minas Gerais não disponibilizou dados referentes ao período 2020.2.

Diversos indicadores socioeconômicos apresentaram associações significativas com a prevalência de HIV em pessoas privadas de liberdade no Brasil. O IDHM mostrou uma associação negativa significativa (p-valor 0,027), indicando que um aumento no IDHM está associado a uma redução na prevalência de HIV. 

A taxa de envelhecimento exibiu uma associação positiva significativa com a prevalência de HIV (p-valor 0,014), indicando que um aumento na taxa está associado a um aumento na prevalência de HIV. 

O percentual de vulneráveis à pobreza apresentou uma associação positiva, mas sem significância estatística (p-valor 0,056), sugerindo que um aumento na proporção de pobres pode estar associado a um aumento na prevalência de HIV. 

A renda domiciliar per capita também mostrou uma associação positiva significativa com a prevalência de HIV (p-valor 0,021), indicando que um aumento na renda domiciliar per capita está associado a um aumento na prevalência de HIV. Em contrapartida, a taxa de analfabetismo para pessoas com 25 anos ou mais apresentou uma associação negativa significativa (p-valor 0,035), sugerindo que um aumento na taxa de analfabetismo está associado a uma redução na prevalência de HIV.

Por fim, o IDHM ajustado para renda mostrou uma associação positiva significativa com a prevalência de HIV (p-valor 0,024), indicando que um aumento no IDHM ajustado para renda está associado a um aumento na prevalência de HIV (Tabela 3).

**Tabela 3:** Associação entre a prevalência do HIV em pessoas privadas de liberdade e indicadores socioeconômicos. Brasil, 2017-2023

Indicadores socioeconômicos	Coeficiente	Erro	p-valor
Índice de desenvolvimento humano municipal	-186,67	77,76	0,027
Taxa de envelhecimento	1,28	0,47	0,014
Percentual de vulneráveis à pobreza	0,74	0,36	0,056
Renda per capita	0,02	0,01	0,021
Taxa de analfabetismo^a^	-0,60	0,26	0,035
Renda ajustada à desigualdade	143,92	58,45	0,024

^a^25 anos ou mais de idade.

## Discussão

Entre 2017 e 2023, a prevalência de HIV em pessoas privadas de liberdade no Brasil revelou padrões marcantes de desigualdade por sexo, região e unidade federativa, além de associações com indicadores socioeconômicos. A taxa de prevalência foi maior entre mulheres, embora a tendência ao longo do período fosse decrescente, em contraste com o aumento observado na tendência entre homens.

As regiões Sul e Sudeste registraram as maiores prevalências, com destaque para o Rio Grande do Sul. Contudo, a análise das tendências apontou variações significativas entre regiões e estados: Pará e Rio de Janeiro apresentaram aumento, enquanto Paraná e Pernambuco tiveram redução.

Cabe ressaltar que a prevalência de HIV no ambiente prisional é maior quando comparada à população geral (21). Nesse contexto, além do cenário insalubre e superlotado desses ambientes, o perfil sociodemográfico das pessoas privadas de liberdade favorece o aumento da vulnerabilidade ao HIV e demais doenças infectocontagiosas (6).

A prevalência da infecção por HIV no ambiente prisional reflete diversos fatores estruturais e sociais, como a falta de acesso adequado a cuidados de saúde e as condições precárias de vida dentro das prisões, que facilitam a disseminação do vírus (22). Além disso, o ato sexual inseguro, principalmente por violência, o uso de drogas e compartilhamento de material cortante estão entre os principais riscos determinantes para as necessidades de saúde desta população (6). 

Ademais, a estigmatização e a marginalização deste público podem dificultar o acesso a programas de prevenção e tratamento, ampliando sua vulnerabilidade ao HIV (23). A disseminação do HIV em estabelecimentos prisionais também está intimamente ligada a políticas eficazes de saúde e intervenções específicas que abordem as particularidades desse contexto nas diferentes regiões do (24).

Verificou-se uma maior taxa de prevalência de HIV no Brasil entre mulheres privadas de liberdade, em comparação aos homens, o que sugere que as mulheres enfrentam desafios específicos e mais complexos no ambiente prisional. Diante disso, uma combinação de fatores de vulnerabilidades pode estar relacionada, não apenas à falta de acesso à informação sobre os métodos de prevenção e transmissão, mas também a exposição à violência sexual, percepções distorcidas de risco ou dificuldade em obter preservativos e assistência de profissionais de saúde (13,25). A alta prevalência também pode refletir subdiagnóstico em períodos anteriores, maior rastreamento recente e impacto da unidade prisional como local de alta transmissão.

Entretanto, mesmo com a alta taxa de prevalência de HIV no Brasil em mulheres no sistema prisional, a tendência dessa prevalência apresentou-se de forma decrescente ao longo do período de tempo analisado neste estudo. Frente a isso, destaca-se a Política Nacional de Atenção às Mulheres em Situação de Privação de Liberdade e Egressas do Sistema Prisional, promulgada no ano de 2014, voltada a atender as especificidades das mulheres e garantir humanização das condições do cumprimento da pena, embora estudos evidenciem falta de efetividade no cumprimento dessa política, considerando que barreiras institucionais, superlotação e discriminação afetam o direito à saúde dessas mulheres (26-27).

Este estudo revelou uma maior prevalência de HIV nas regiões Sul e Sudeste do Brasil. Apesar do desenvolvimento econômico destas regiões, populações em situação de vulnerabilidade podem enfrentar barreiras no acesso a cuidados de saúde adequados. A alta densidade populacional em ambientes urbanos, especialmente em áreas de baixa renda, pode levar a práticas de risco, como o uso de drogas injetáveis e relações sexuais desprotegidas, fatores de risco para a transmissão do HIV (28). 

No período de estudo avaliado, as tendências da prevalência de HIV na população privada de liberdade variaram entre regiões e estados. No Sudeste, observou-se um aumento da tendência, principalmente, entre os homens. Tais resultados são concordantes com dados de estudo sobre a prevalência de aids na região sudeste, no qual se identificou um predomínio da infecção no público masculino, destacando-se os estados do Rio de Janeiro e São Paulo (29).

Em contrapartida, estados como Pernambuco e Paraná apresentaram tendência de queda na prevalência de HIV, podendo indicar avanços no enfrentamento da doença. Assim, ao identificar detentos soropositivos para HIV é possível formular estratégias de prevenção, como a ampliação dos testes rápidos e educação em saúde, além do acesso adequado aos serviços de saúde para o tratamento e acompanhamento de forma contínua, o que podem ter contribuído para a diminuição da transmissão do vírus entre a população carcerária nesses estados (30). 

A expansão do acesso ao diagnóstico de HIV mediante testes rápidos favorece a detecção precoce e o tratamento oportuno, além de que as articulações com a rede de atenção à saúde são essenciais para ofertar um cuidado integral e contribuir na supressão da carga viral das pessoas que vivem com HIV no sistema prisional (23). Tais medidas, quando efetivadas, podem explicar o padrão estacionário da tendência de prevalência de HIV na maioria dos estados e regiões brasileiras. 

Observou-se ainda uma associação entre o aumento do IDHM e a redução da taxa de infectividade pelo HIV entre indivíduos privados de liberdade. O IDHM é uma medida que analisa o desenvolvimento humano de um município no quesito renda, escolaridade e saúde. De fato, análises de dados secundários globais, fornecidos pelo Global Burden of Disease Study, indicam que entre 1990 e 2019, regiões com maior desenvolvimento socioeconômico apresentaram queda nessas taxas (31). Em consonância, entre pessoas privadas de liberdade, observa-se que em cidades da África do Sul, país considerado epicentro (32) da epidemia de HIV/aids, a incidência de casos de soropositividade para o HIV tem aumentado e apresenta prevalência de 17,7% (33); enquanto, na Europa, em cidades de países como França e Itália, a prevalência varia de ≥2% a <5%, respectivamente (34).

No entanto, a relação entre IDHM, renda e prevalência do HIV podem variar espacial e temporalmente em um país devido às diferenças por regiões, o qual pode exercer associação positiva ou negativa, independentemente do contexto desta ser favorável ou não (35). Se tratando da população privada, observou-se que esse cenário também ocorre. Neste estudo, por exemplo, o aumento da renda nacional, o aumento no IDHM ajustado para renda e o aumento da proporção de indivíduos pobres estão associados ao aumento na prevalência do HIV em pessoas privadas de liberdade. 

Especificamente sobre a renda, entende-se que não é viável estabelecer uma comparação entre a situação econômica da população privada de liberdade e a soropositividade ao HIV, pois, ao participarem de pesquisas, muitos optam por não informar sua renda, temendo que essa informação possa ser vinculada ao crime ou delito cometido, além do que, o sistema de Levantamento Nacional de Informações Penitenciárias não disponibiliza dados sobre a renda da população carcerária no Brasil (28). 

A relação entre o aumento de indivíduos em situação de pobreza e a incidência de HIV pode estar relacionada a comportamentos sexuais de risco. Estudo realizado no Malawi, país africano com um dos menores Índices de Desenvolvimento Humano (IDH) do mundo (0,0512) (36) e prevalência de HIV superior a 23% entre a população privada de liberdade (37), revelou que a pobreza levou alguns detentos a se envolverem em atividades sexuais em troca de benefícios, como espaço para dormir e melhores alimentos, fornecidos por prisioneiros com penas mais longas (38).

A taxa de analfabetismo em pessoas com 25 anos ou mais de idade apresentou relação negativa com a prevalência de HIV, sugerindo que estados com mais pessoas que não sabem ler ou escrever, registram menos casos de HIV. Contudo, acredita-se que esta associação pode refletir subnotificação de dados, devido à baixa adesão a estratégias de diagnóstico entre esse público, uma vez que, ao possuírem baixo letramento em saúde, podem desconhecer as medidas de diagnóstico do HIV, o que pode influenciar na realização do exame (39).

Além disso, o aumento da taxa de envelhecimento na crescente prevalência do HIV entre a população carcerária pode estar ligado à maior adesão aos testes de rastreio do HIV, refletindo maior conscientização dos riscos do HIV entre pessoas mais velhas, quando comparadas a jovens privados de liberdade (39-40).

Como limitação do estudo, destaca-se a procedência dos dados analisados, obtidos em um sistema que pode estar sujeito a subnotificações e inadequação dos registros, o que pode comprometer a qualidade das informações coletadas. A subnotificação pode ocorrer devido à ausência de testagem sistemática e dificuldades no registro adequado dos casos, impactando a real dimensão da prevalência do HIV nessa população. 

Além disso, a utilização de dados oriundos do sistema penitenciário, em detrimento de outras bases, como o Sinan, pode não abranger todas as unidades prisionais do país de maneira uniforme, especialmente em estados ou regiões com menor capacidade de gestão penitenciária. Esse sistema pode não integrar informações prévias ou posteriores ao período de privação de liberdade, como diagnósticos realizados em unidades de saúde externas, limitando a análise da trajetória clínica das pessoas privadas de liberdade. 

Outra limitação importante decorre do delineamento de série temporal, que permite identificar padrões e tendências ao longo do tempo, mas não possibilita estabelecer relações de causalidade nem extrapolações individuais. A interpretação dos resultados deve considerar esses aspectos, e pesquisas futuras podem aprofundar essas análises com abordagens metodológicas complementares.

Este estudo evidenciou variações significativas na tendência temporal da prevalência do HIV por sexo, região e estado entre a população brasileira privada de liberdade. Nacionalmente, a prevalência foi maior entre as mulheres, mas apresentou tendência de redução ao longo do período analisado, enquanto entre os homens houve aumento. Nas regiões, o Sudeste, teve aumento da prevalência do HIV entre os homens, enquanto o Nordeste registrou redução entre as mulheres. Em estados como Rio de Janeiro e Pará, a prevalência aumentou entre os homens, enquanto no Paraná e em Pernambuco, a tendência da prevalência de queda foi maior entre as mulheres. A queda do número de casos entre as mulheres e do crescimento entre os homens indica a necessidade de ações eficazes e direcionadas por meio da implementação de estratégias preventivas e de tratamento entre o público masculino, no qual devem ser organizadas estrategicamente, considerando o tamanho desta população.

As descobertas sobre os determinantes socioeconômicos, como o índice de desenvolvimento humano municipal ajustado para a renda, a renda domiciliar per capita e as taxas de analfabetismo, mostraram-se associados à prevalência do HIV, ressaltando a influência de desigualdades estruturais. Estes resultados apontam para a complexidade do cenário e a necessidade de abordagens integradas que combinem ações de saúde pública com iniciativas de redução de desigualdades sociais.

O apontamento deste panorama, pode contribuir, portanto, para a modulação estratégica de ações de saúde, indicando que o planejamento destas, deve ser pautado nas disparidades regionais e de gênero, e, que, devem considerar os determinantes socioeconômicos para formular suas intervenções para a redução da prevalência do HIV em pessoas privadas de liberdade no Brasil.

## Data Availability

O banco de dados utilizado na pesquisa está disponível em https://osf.io/shn7k/.
